# Arteriolar neuropathology in cerebral microvascular disease

**DOI:** 10.1111/nan.12875

**Published:** 2023-01-29

**Authors:** Chuo Fang, Shino D. Magaki, Ronald C. Kim, Raj N. Kalaria, Harry V. Vinters, Mark Fisher

**Affiliations:** ^1^ Department of Neurology University of California, Irvine Medical Center 101 The City Drive South Shanbrom Hall (Building 55), Room 121 Orange 92868 California USA; ^2^ Department of Pathology & Laboratory Medicine, David Geffen School of Medicine University of California, Los Angeles Los Angeles California USA; ^3^ Department of Pathology & Laboratory Medicine University of California, Irvine Orange California USA; ^4^ Translational and Clinical Research Institute Newcastle University Campus for Ageing and Vitality Newcastle upon Tyne NE4 5PL UK; ^5^ Department of Neurology, David Geffen School of Medicine University of California, Los Angeles Los Angeles California USA

**Keywords:** cerebral angiomyopathy, cerebral arterioles, cerebral microvascular disease, neuropathology, smooth muscle cells

## Abstract

Cerebral microvascular disease (MVD) is an important cause of vascular cognitive impairment. MVD is heterogeneous in aetiology, ranging from universal ageing to the sporadic (hypertension, sporadic cerebral amyloid angiopathy [CAA] and chronic kidney disease) and the genetic (e.g., familial CAA, cerebral autosomal dominant arteriopathy with subcortical infarcts and leukoencephalopathy [CADASIL] and cerebral autosomal recessive arteriopathy with subcortical infarcts and leukoencephalopathy [CARASIL]). The brain parenchymal consequences of MVD predominantly consist of lacunar infarcts (lacunes), microinfarcts, white matter disease of ageing and microhaemorrhages. MVD is characterised by substantial arteriolar neuropathology involving ubiquitous vascular smooth muscle cell (SMC) abnormalities. Cerebral MVD is characterised by a wide variety of arteriolar injuries but only a limited number of parenchymal manifestations. We reason that the cerebral arteriole plays a dominant role in the pathogenesis of each type of MVD. Perturbations in signalling and function (i.e., changes in proliferation, apoptosis, phenotypic switch and migration of SMC) are prominent in the pathogenesis of cerebral MVD, making ‘cerebral angiomyopathy’ an appropriate term to describe the spectrum of pathologic abnormalities. The evidence suggests that the cerebral arteriole acts as both source and mediator of parenchymal injury in MVD.

## Graphical abstract

Cerebral microvascular disease (MVD) is an important cause of vascular cognitive impairment, characterised by differential arteriolar pathology involving ubiquitous vascular smooth muscle cell abnormalities. These include changes in proliferation, migration, phenotypic switches and delayed cell death mechanisms. The cerebral arteriole is a key target, which undergoes ‘cerebral angiomyopathy’ acting as both the source and mediator of parenchymal injury in MVD.

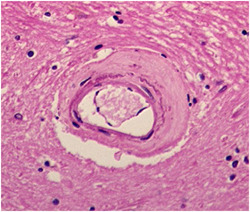

**Key points**

- Cerebral microvascular disease (MVD) has a wide range of aetiologies ranging from universal (ageing) to sporadic and genetic.
- Brain parenchymal consequences of MVD consist of lacunar infarcts (lacunes), microinfarcts, white matter disease/demyelination and microhaemorrhages.
- Abnormalities in arterioles are the final common pathway in producing the observable brain parenchymal consequences.
- The term *cerebral angiomyopathy* describes this spectrum of pathologic abnormalities of the arteriolar wall.

## INTRODUCTION

Cerebral microvascular disease (MVD) refers to a group of conditions that affect small vessels of the brain; they are heterogeneous in their aetiology, and with variable, though often profound, consequences for brain function. MVD is a major public health issue and is an important cause of vascular cognitive impairment [1, 2]. Despite its high prevalence, there remain critical unanswered questions regarding its pathogenesis that have resulted in suboptimal treatment efforts and preventive strategies.

The range of MVD entities is well‐described. It is encountered universally in ageing and arises sporadically such as in hypertension, sporadic cerebral amyloid angiopathy (CAA) and chronic kidney disease (CKD); it also occurs in genetic forms including familial CAA, cerebral autosomal dominant arteriopathy with subcortical infarcts and leukoencephalopathy (CADASIL), cerebral autosomal recessive arteriopathy with subcortical infarcts and leukoencephalopathy (CARASIL), Fabry disease, cerebroretinal vasculopathy (CRV, or retinal vasculopathy with cerebral leukodystrophy [RVCL]), collagen IV (COL4) mutations and cathepsin A‐related arteriopathy with stroke and leukoencephalopathy (CARASAL). While this range is substantial, the parenchymal consequences of MVD are limited and consist of lacunar infarcts (lacunes), variably defined as grossly visible cystic lesions less than 1 to 2 cm in greatest diameter [2]; microinfarcts, ischaemic lesions visible only on microscopic examination [2]; white matter disease of ageing [2, 3]; and microhaemorrhages [4, 5]. Our review addresses the incongruity of MVD, given the wide range of provoking factors versus the relatively limited range of neuropathologic manifestations that affect the brain. It is argued that the cerebral arteriole plays a dominant role in MVD pathogenesis. On one hand, there exists a range of arteriolar pathologic changes that are relatively distinct for each entity. At the same time, these distinctive changes substantially reflect abnormalities of the arteriolar wall characterised by alterations of arteriolar smooth muscle cells (SMC) and lead to occlusion or rupture of cerebral blood vessels. The term *cerebral angiomyopathy* has been proposed to describe this phenomenon [6]. The parenchymal consequences of these heterogeneous arteriolar changes are limited, suggesting that the abnormalities of cerebral arterioles function as a final common pathway for these structural changes.

## CEREBRAL ARTERIOLAR NETWORK AND COMPOSITION

The arteriolar alterations in MVD may lead to common parenchymal consequences, including lacunar infarcts (lacunes), microinfarcts, white matter disease/demyelination and microhaemorrhages. Cerebral arterioles are particularly important given their role as the major site of vascular resistance and primary regulators of cerebral blood flow [7]. The cerebral arteriolar network is divided into three major components: (1) pial arterioles on the surface of the brain; (2) parenchymal/penetrating arterioles that branch out from pial arterioles to enter the brain parenchyma or arise from deep penetrating arteries in the basal ganglia, thalamus and brainstem; and (3) downstream pre‐capillary arterioles [8, 9]. The walls of cerebral arterioles, which typically have a diameter in the range of 10–100 μm [10], consist of three layers: tunica intima, tunica media and tunica adventitia [11]. The tunica intima is composed of a single layer of endothelial cells and the basal lamina surrounding endothelium. Endothelial cells in the intima are vital in the regulation of vascular tone by releasing vasoactive factors such as nitric oxide, prostacyclin, thromboxane and endothelin‐1 [12], which regulate the contractile state of SMC and ultimately vascular diameter. The tunica media in arterioles consists of one or two complete layers of square‐ or rectangular‐shaped SMC with surrounding elastin and collagen fibres [13]. In larger arterioles with a diameter less than 100 μm, but larger than 40 μm [14], the media may be separated from the innermost intima by the internal elastic lamina. The SMC in the media contract to change the vascular diameter and regulate blood flow between the arteries and capillaries. The tunica adventitia is comprised primarily of collagen fibres and fibroblasts; it provides structural support for the vascular wall and is involved in repair of the vessel wall following injury [15].

Given the complex structure of arterioles and the critical role of each component, the correct identification of cerebral arterioles is of great importance in understanding the pathology of MVD and the development of targeted preventative and treatment strategies. Traditionally, arterioles are distinguished from veins/venules on the basis of smooth muscle content, the shape of the vessel and the presence/absence of internal elastic lamina. Unlike arteries/arterioles, veins and venules normally have minimal SMC and do not have an elastic lamina [16]. However, small arterioles and venules in the brain parenchyma have similar intramural cell types, that is, SMC and pericytes in the media; both SMC and pericytes contain variable amounts of smooth muscle actin. Pericytes are embedded within the basement membrane of capillaries and play a vital role in blood–brain barrier (BBB) maintenance and cerebral blood flow control. Increase in pericyte numbers in the walls of arterioles of patients with CADASIL is linked to SMC degeneration and vessel wall thickening [17]. Beyond that, the substantial overlap in lumen diameter to wall thickness ratio between arterioles and venules challenges traditional methods that use light microscopy to distinguish arterioles from venules; the sclerotic index is a quantified assessment that may assist with this distinction [18, 19, 20].

## SMOOTH MUSCLE CELLS (SMC) IN VASCULAR REMODELLING

Current understanding of the pathogenic mechanisms underlying MVD is limited due to the challenges in visualising the diseased small vessels via radiographic imaging in vivo. SMC integrity is necessary for cerebrovascular health owing to their principal role in vascular wall contraction and remodelling, involving the phenotypic switch of SMC from the quiescent, contractile phenotype to the proliferative, migratory phenotype. Perturbations in SMC signalling and function are suggested to underlie the pathogenesis of cerebral microangiopathies, in particular hypertensive microangiopathy, CAA, CADASIL [21], Fabry Disease [22] and CRV/RVCL [23]. The function of other vascular cell components, for example, endothelial cells, can also be compromised with ageing, hypertension and other risk factors [24]. Important functions of SMC in diseased conditions include apoptosis, phenotypic switch, extracellular matrix degradation, proliferation and contractility. Inflammatory cell infiltration and genetic changes may modulate SMC functions and involve changes in growth factor signalling and regulation of RNA expression. One of the key factors that has been highlighted recurrently in recent years is transforming growth factor‐β (TGF‐β), with particular reference to hereditary MVD of the brain [25]. TGF‐β isoforms are upregulated and activated in vascular diseases and have an important role in muscle repair and remodelling, as well as regulation and function of myocytes, fibroblasts, immune cells and other vascular cells. Although dysregulation of TGF‐β is not primary in all MVD, nor is it the only molecular mechanism underlying MVD arteriolosclerosis, the contribution of TGF‐β and vascular basement membrane disruption in the pathogenesis of MVD is clear [25].

The morphologic changes in the vasculature, such as vessel wall disruption and fibrosis, may be highlighted using special stains and immunohistochemistry. These include Verhoeff‐Van Gieson staining (for elastin), Masson's trichrome staining (for collagen) [26], α‐smooth muscle actin immunohistochemistry (for SMC), Prussian blue (for iron, including haemosiderin) [18], periodic acid‐Schiff (PAS) stain (suggesting the presence of granular material within vessel walls and highlighting basement membrane components) [27] and Richardson's staining (for globotriaosylceramide‐3) [28]. These histologic and classical tinctorial stains such as PAS and Oil‐Red‐O enable one to identify some common features of the changes occurring in arterial walls in MVD.

## AGEING

Ageing is associated with brain atrophy and lesions such as lacunes, white matter hyperintensities (WMH) and cerebral microbleeds (CMB) as evident on magnetic resonance imaging (MRI) [29]. Ageing is considered an independent risk factor for vascular dysfunction. The best‐described arteriolar changes with ageing, primarily in pial arterioles, include a significant decrease in arteriolar number [7] and increase in arteriolar diameter [30]. Increasing age is associated with medial elastin loss with replacement by collagen, gradual intimal thickening evident by a decrease in lumen diameter and an increase in intima‐to‐media thickness and adventitial fibrosis. These age‐related structural changes contribute to mechanical alterations such as reduced compliance and elasticity and increased arterial stiffness [24, 30]. Recent studies in mice suggest ageing is associated with increased wall thickness but reduced wall stress in parenchymal arterioles, which is a potential mechanism to protect these arterioles from vascular injury. Loss of myogenic tone in large arteries with age may increase the risk of rupture of parenchymal arterioles with blood pressure fluctuations [31]. However, ageing is not associated with changes in distensibility or lumen diameter in parenchymal arterioles [31, 32].

Impairment of vascular structure and function with ageing is largely driven by multiple mechanisms involving changes in SMC number, either by affecting their proliferation or by cell death, predominantly by apoptosis. A large number of in vitro [33, 34, 35, 36, 37, 38, 39] and in vivo [34] studies have demonstrated that ageing is associated with increased SMC proliferation, as evidenced by increased expression of stem cell markers [33] and cell cycle activation markers [34] in SMC from humans and rodents. However, conflicting results have shown a loss of proliferation of SMC isolated from aged human donors [40, 41] and rodent models [42, 43]. The proliferation rate of SMC in each passage in culture is dependent on donor age, with SMC from aged donors more likely to be senescent with impaired proliferative capacity [40]. Decrease in SMC number with advanced age can also be a result of increased apoptosis [34, 43].

Vascular SMC have extended life expectancy, and the effect of age on SMC is complex. SMC may initially undergo hyperproliferation early in the ageing process. With advanced age, SMC gradually exhibit cellular senescence, characterised by impaired proliferative capacity, irreversible growth arrest and apoptosis; this contributes to vascular inflammation, loss of arterial function and development of age‐related disease [44]. In addition, differences in animal models, experimental conditions and patient comorbidities (among other factors) are likely to underlie the conflicting results in studies of age‐related changes in SMC.

Another underlying mechanism relates to phenotypic alterations of SMC. SMC are normally quiescent and contractile to maintain vascular tone. With ageing, they adopt a stiff and pro‐migratory phenotype. Aged SMC have an accelerated cell cycle and increased reactive oxygen species (ROS) production compared with their younger counterparts. Aged SMC produce more matrix metalloproteinases (MMP) that promote SMC migration from the media to the intima by detaching cells from the extracellular matrix (ECM); this process contributes to elastin fragmentation and loss with replacement by collagen and resultant arteriolar wall stiffening [24].

Together, these changes in SMC (increase or decrease in proliferation and migration) are key events in ageing that lead to vessel wall thickening and stiffening and to vascular dysfunction that may contribute to age‐related MVD. It should be noted that age‐related CAA may lead to increased fragility of the vessel wall rather than stiffening, making the vessels prone to bleeding [45]. Animal models allow us to study ageing as an independent risk factor; in humans, age‐related vascular remodelling increases the risk of MVD when other risk factors are present, in particular hypertension. Hence, it is very difficult to isolate the effects of ageing from hypertension on the cerebral arterioles.

## HYPERTENSION

**FIGURE 1:**
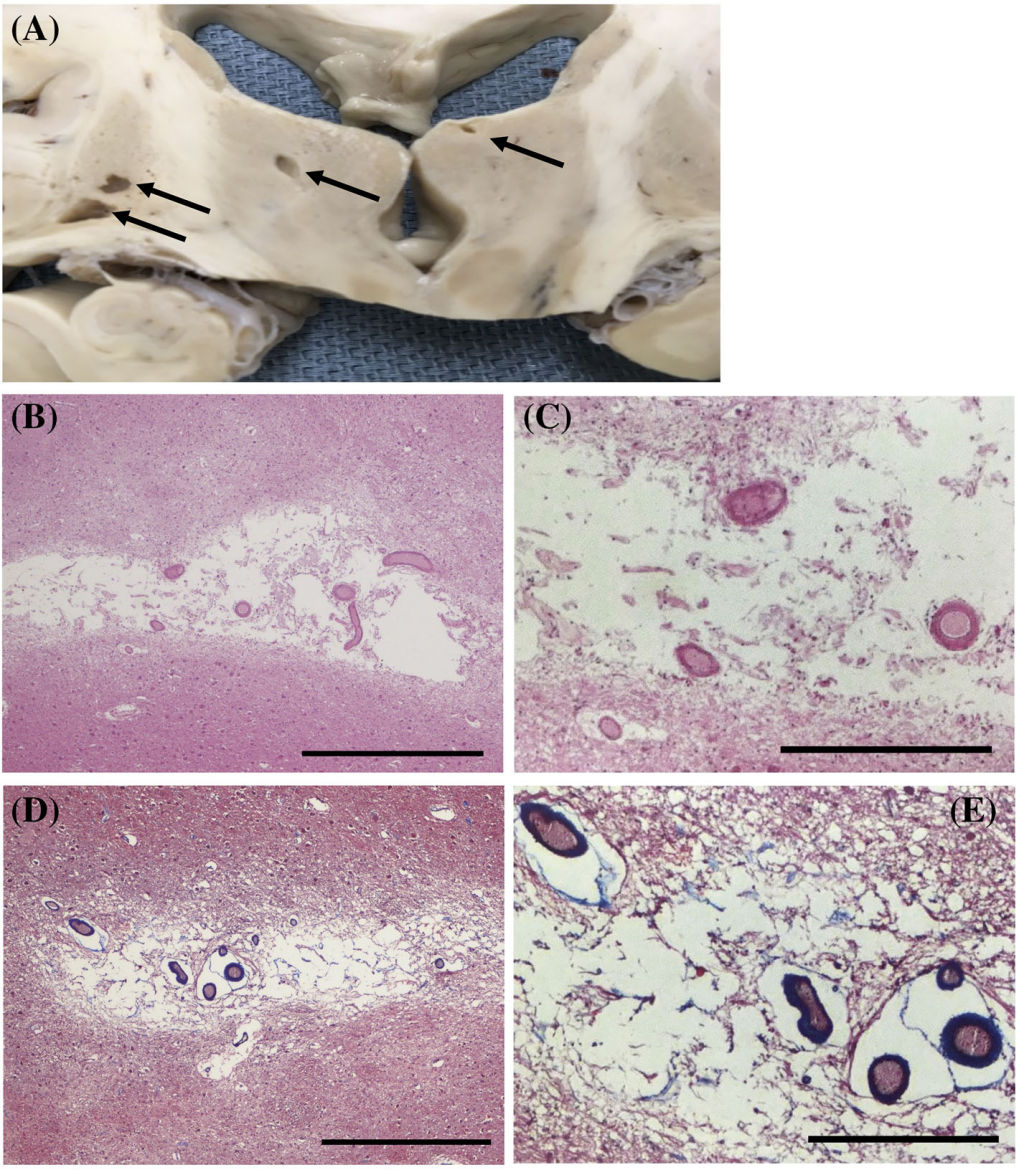
Arteriolar neuropathology in hypertension. (A) Lacunar infarcts (arrows) in the basal ganglia and thalamus of an autopsy specimen. (B, C) Hyalinosis with a ‘glassy’ or hyalinised appearance of the arteriolar wall associated with infarcts in these sections (hematoxylin & eosin [H&E]) and characterised by vessel wall thickening. (D, E) Profound collagen replacement (blue) and smooth muscle cell (SMC) loss and degeneration in the arteriolar wall with hypertension (Masson's trichrome). A normal arteriole would stain red by Masson's trichrome due to the presence of SMC. (B, D) Scale bar 600 μm. (C, E) Scale bar 200 μm

Hypertension is a major risk factor for the development of ischaemic stroke, intracerebral haemorrhage (ICH) and dementia. It is associated with brain atrophy, microinfarcts and microhaemorrhages (Figure 1A), which are important factors in the degree and temporal evolution of cognitive impairment. Hypertension has an especially profound effect on parenchymal/penetrating arterioles and pial arterioles [9]. Parenchymal/penetrating arterioles are particularly significant in hypertension because of their limited collateral circulation, and their dysfunction is likely to cause insufficient blood supply and damage to the deep white matter and grey nuclei [9].

The hallmark of the vascular pathology of hypertension is arteriolosclerosis, which causes thickening of the arteriolar wall with SMC degeneration and loss [19, 46]. Obstruction of the lumen may cause impaired blood flow leading to oligaemia (hypoperfusive state), lacunar infarcts, microinfarcts and WMH. The adventitial layer undergoes ECM remodelling, leading to collagen deposition and thus arteriolar fibrosis [11]. The severity of arteriolosclerosis is closely related to age and can be exacerbated by hypertension [47]. In hypertension, the affected vessel wall (typically less than 150 μm in diameter) can develop a ‘glassy’ hyalinized and/or an ‘onion‐skinning’ hyperplastic appearance as a result of degenerated SMC and elastin in the media and proliferated fibroblasts in the adventitia [2] (Figure 1B–E). Fibrinoid necrosis, due to the infiltration of fibrin and fibrin degradation products in the vessel wall, is more common in malignant hypertension and may be seen with severe CAA. Fibrinoid necrosis in subcortical penetrating arterioles causing weakening of the arteriolar wall is likely an underlying cause of ICH in patients with severe hypertension. In patients with severe CAA, it is probably a major contributing factor to cerebral haemorrhage. Hyalinosis and fibrinoid necrosis can be distinguished from each other as hyaline material contains only degenerated SMC and collagen and no fibrin is present [48]. (To avoid confusion, we do not use the term ‘lipohyalinosis’ [48].) While the exact cellular rearrangements are unclear, gradual hypertension‐related changes in the arteriolar wall lie within a spectrum and depend on the severity of other perivascular influences. Other pathological consequences of hypertension include microatheroma (distal manifestations of atherosclerosis involving larger arterioles) and microaneurysms (segmental dilatation of vessels) [9, 49]. The proliferation of mural cells may exert secondary effects that raise vascular pressure and promote SMC degeneration in the upstream feeding arterioles, thereby inducing arteriolar wall rupture and subsequent haemorrhage [50].

## CEREBRAL AMYLOID ANGIOPATHY (CAA)

**FIGURE 2:**
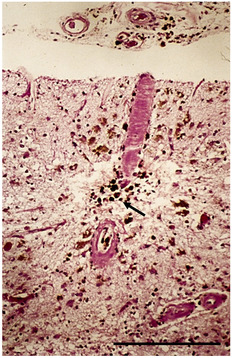
Arteriolar cerebral amyloid angiopathy (CAA). Arachnoid membrane and subarachnoid space above superficial right parieto‐occipital cortex, with amyloid‐laden arterioles associated with haemosiderin‐laden macrophages (arrow) (hematoxylin & eosin [H&E]). Scale bar 1200 μm

CAA is a common MVD associated with Alzheimer's disease [51, 52] and is often observed in the brains of the elderly even in the absence of Alzheimer's disease. CAA is characterised by the accumulation of amyloid‐β (Aβ) in the walls of small‐to‐medium‐sized arteries and arterioles predominantly located in the leptomeninges and cerebral cortex; these are prone to bleeding due to the replacement of the medial SMC with Aβ and fragility of the affected arteries/arterioles [53]. CAA is an important cause of spontaneous primary lobar haemorrhage in elderly individuals and is also associated with microinfarcts [53, 54], microhaemorrhages and superficial siderosis [55]. The spatial distribution of cerebral microhaemorrhages in the brain correlates well with the anatomic distribution of affected small vessels. CMB located in cortical and subcortical regions is considered a surrogate marker of CAA on imaging, and cortical CMB are particularly associated with severe CAA [56]. Figure 2 demonstrates an amyloid‐laden penetrating arteriole with surrounding haemosiderin‐laden macrophages in the parenchyma, consistent with prior haemorrhage. CAA preferentially affects arterioles as they are postulated to be the main routes for perivascular Aβ clearance [57].

Post‐mortem examination of human tissue [58] and several transgenic mouse models [59, 60] of CAA overexpressing mutant human Aβ precursor protein (APP) have shown sequential arteriolar wall changes. The endothelial layer is relatively preserved, whereas the SMC layer is increasingly disrupted with progressing CAA severity. Soluble Aβ‐induced functional abnormalities such as failure to respond to vasoactive stimuli are early manifestations despite little or no CAA vascular pathology [61]. In the early stages or mild CAA, Aβ is deposited in the external basal lamina in close proximity to the SMC layer and in the adventitia, but with no SMC replacement or disruption [62]. As the disease progresses, Aβ is deposited in the SMC layer, which is disrupted, and the internal basal lamina appears thinner and irregular [61, 62]; this is followed by SMC loss and complete replacement by Aβ [59, 60]. Advanced CAA is also associated with the formation of microaneurysms and fibrinoid necrosis in Aβ‐laden vessels; these are often associated with cerebral lobar haemorrhage [62].

The vascular origin of microhaemorrhages in the context of CAA is complex and remains poorly understood. There are fewer arterioles with Aβ deposition immediately surrounding microhaemorrhages, compared with areas with microinfarcts [63]. Aβ is more frequently observed upstream or downstream from the site of rupture, indicating that microhaemorrhages may occur at a later time point of the disease [63]. Arterioles associated with microhaemorrhages are more likely to be degenerated, allowing extravasation of red blood cells. In regions of microinfarcts, the arterioles appear to be more intact, suggestive of a perfusion‐mediated phenomenon resulting in infarcts [63]. The spatial relationship between microhaemorrhages and vascular Aβ deposition has been assessed in CAA mice and their wild‐type littermates. Rather than originating from CAA‐laden vessels, vascular segments without Aβ deposition appeared vulnerable to progressive vessel wall weakening and more prone to bleeding. Leakage sites were most likely to be branch points of penetrating arterioles, but capillaries (BBB leakage) could not be excluded [64].

Familial CAA is rare, generally more severe than sporadic CAA and associated with an earlier age of onset and/or death. Familial CAA is commonly present in the form of autosomal dominant disorders, characterised by mutations in the APP [52], cystatin C3 (*CST3*) [65] or integral membrane protein 2B [66] genes, leading to the aggregation of Aβ in blood vessel walls. It has been demonstrated that the pathology of familial CAA is age‐related and results in both cerebral infarcts and ICH. Beyond leptomeningeal and cortical arteries and arterioles, the affected brain regions in familial CAA are thought to be more extensive and involve the cerebellum and brainstem. In rare cases of patients with severe CAA, profound Aβ deposition is seen in the walls of leptomeningeal vessels but less prominent in parenchymal vessels. The associated neuropathological change features cerebral infarcts rather than lobar haemorrhage as a result of wall thickening and occlusion of large‐sized leptomeningeal arteries [53].

## CHRONIC KIDNEY DISEASE (CKD)

**FIGURE 3:**
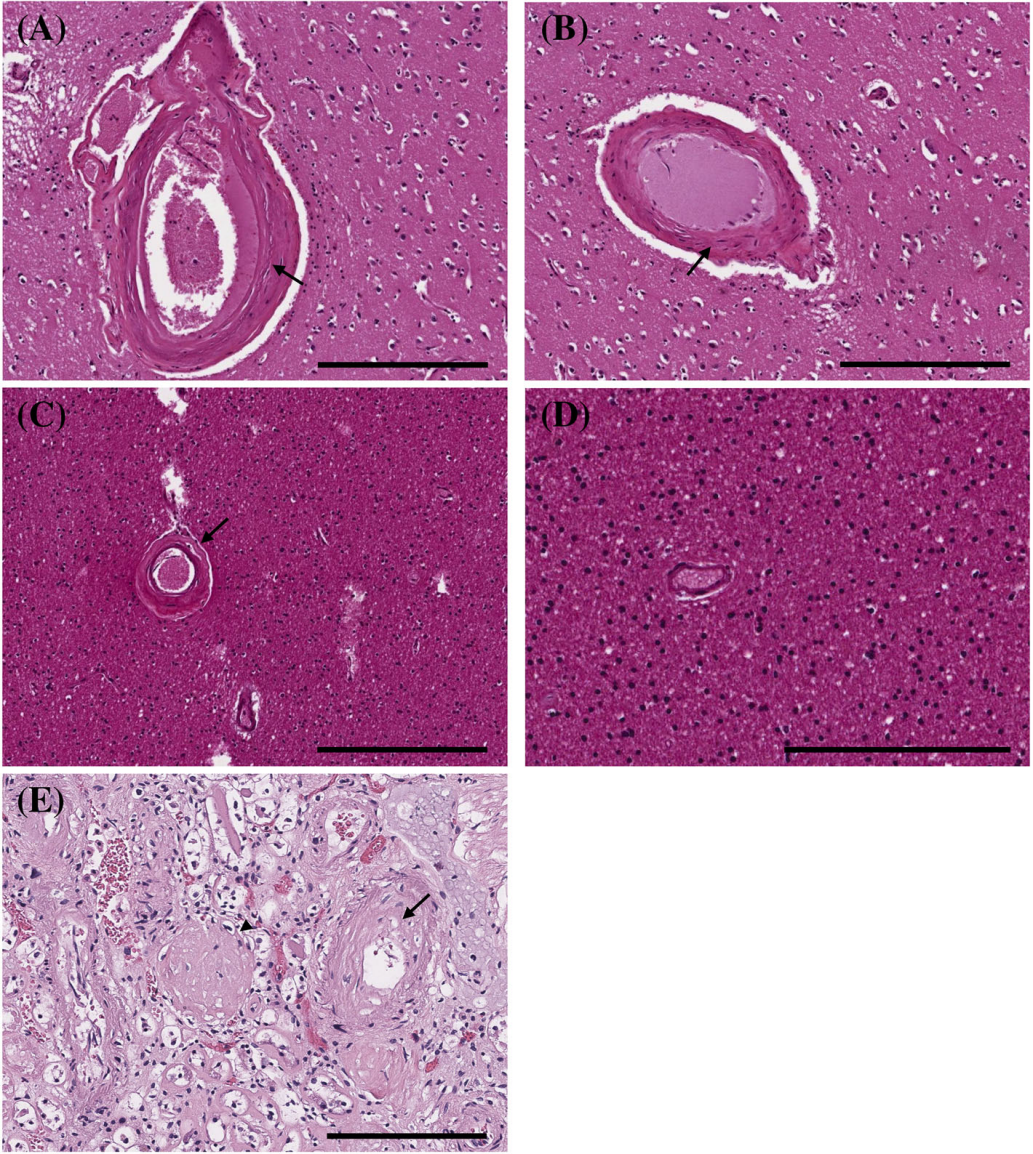
Arteriolar thickening in chronic kidney disease (CKD) I. (A, B) Arteriolosclerotic changes with luminal narrowing, intimal thickening and degeneration of smooth muscle cells (SMC) and elastin in the media in arterioles of the basal ganglia and (C) the white matter. Arrows indicate (A) internal elastic lamina, (B) smooth muscle, and (C) adventitia. (D) Vessel with no/minimal arteriolosclerosis. (E) Kidney from the same patient as panels (A) and (B) showing arteriosclerosis with intimal hyperplasia (arrow) and glomerulosclerosis (arrowhead). (A–C) Scale bar 300 μm. (D, E) Scale bar 200 μm

**FIGURE 4:**
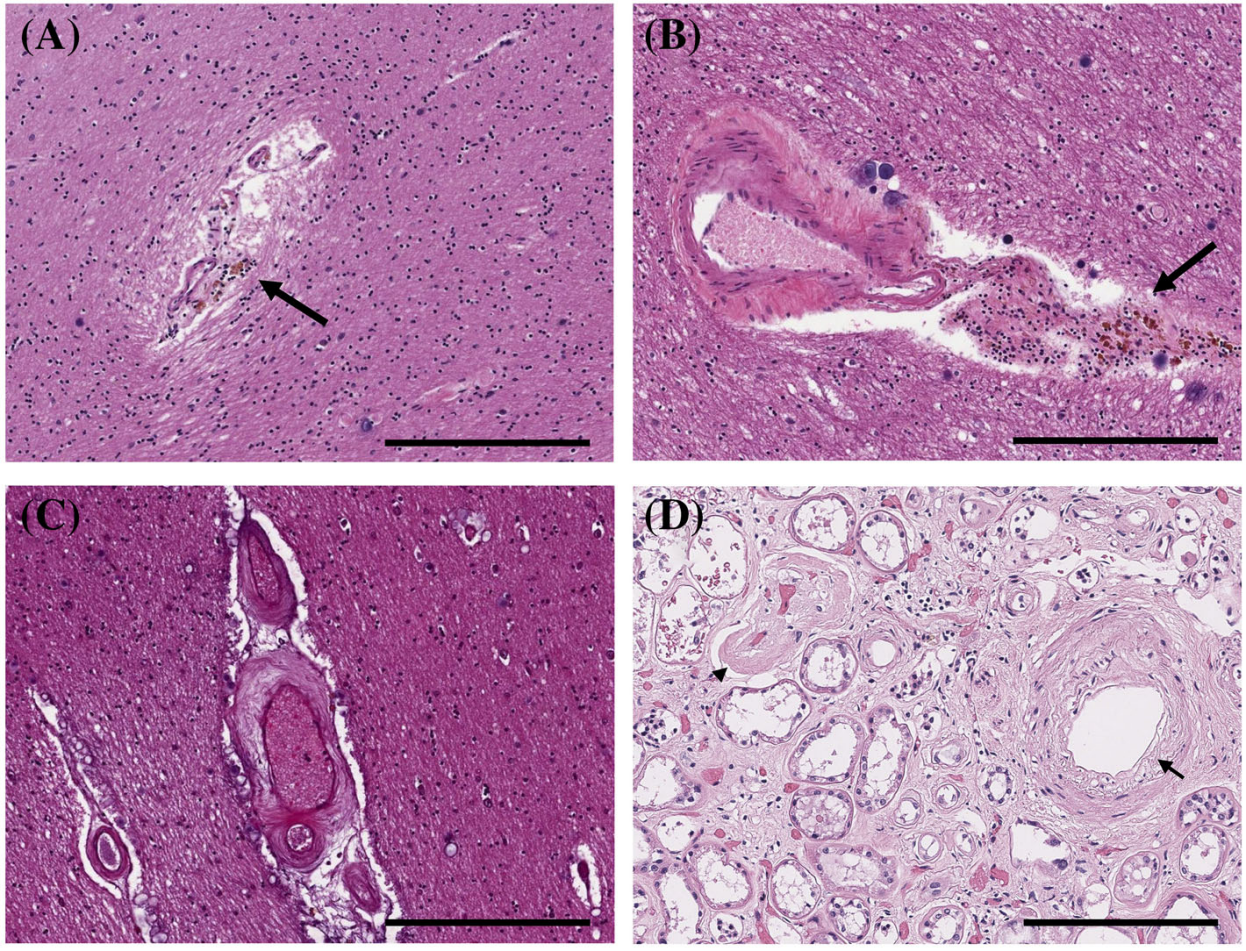
Arteriolar neuropathology in chronic kidney disease (CKD) II. (A, B) Cerebral microhaemorrhages with haemosiderin‐laden macrophages (arrows) adjacent to white matter vessels. (C) Microvascular adventitial fibrosis with marked vessel wall thickening in the subcortical white matter (hematoxylin & eosin [H&E]). (D) Kidney from the same patient as panels (A) and (B) showing hypertensive nephrosclerosis with intimal hyperplasia (arrow) and arteriolar hyalinosis (arrowhead). (A–C) Scale bar 300 μm. (D) Scale bar 200 μm

The kidney and brain share some anatomical and functional similarities; both are characterised by high blood flow rates and dependence on local autoregulation through short, small perforating arterioles. Therefore, the mechanisms underlying the microvascular damage are thought to be similar. Deterioration of these arterioles may markedly accelerate the progression of both renal and cerebral dysfunction. In the kidney, these arterioles, primarily juxtamedullary afferent arterioles, are particularly susceptible to hypertensive injury characterised by hyaline arteriolosclerosis with the replacement of arteriolar SMC by hyaline material. Hyalinosis of deep penetrating arterioles in the brain is associated with lacunar infarction and deep white matter changes and can cause impaired brain function [67] (Figure 3). Another pathological feature of CKD is the presence of haemosiderin‐laden macrophages adjacent to cerebral arterioles, indicating cerebral microhaemorrhages (Figure 4) [68]. However, the co‐occurrence of multiple and heterogenous neuropathologic findings is frequent in CKD patients. Defining the neuropathologic features of CKD is difficult as patients with CKD often have comorbidities such as hypertension that affect the microvasculature.

The relationship between poor kidney function and the development of MVD is not well understood due to the complexity and heterogeneity of the risk factors associated with CKD. A recent investigation has shown that ischaemic infarcts (55%) are the most common neuropathologic change in CKD patients and are associated with arterio/arteriolosclerosis. Less frequently observed were microaneurysms (7.5%) and cerebral haemorrhages (5%). Arteriolosclerotic changes included hyalinization and arterial/arteriolar wall thickening as a result of the deposition of collagen and connective tissues, SMC proliferation and adventitial fibrosis [69].

Two underlying mechanisms leading to MVD in CKD are apoptosis and a shift toward a SMC osteogenic phenotype. In CKD, hyperphosphataemia results from reduced urinary phosphate excretion and continuous intestinal absorption. Initially, exposure of SMC to elevated serum phosphate levels induces apoptosis in SMC, possibly via interrupting energy metabolism in mitochondria [70]. As CKD progresses to late stages, hyperphosphataemia causes further weakening of the vessel wall and arterial calcification by inducing an osteogenic phenotype shift of SMC and elastin degradation in the medial layer [67, 70]. Elevated levels of other minerals in CKD (e.g., calcium) also have synergistic effects on inducing arterial calcification via distinct mechanisms affecting SMC [71].

Arterial (intimal and medial) calcification has been associated with CKD in ischaemic stroke patients [72], as well as in young adults undergoing dialysis [73]. Of note, brain microvascular calcification is frequently seen in CKD patients lacking neurologic deficits, for example, in the basal ganglia (arterioles and capillaries) and hippocampal endplate region (capillaries), and (in a rare case) deep cerebellar white matter (arterioles) [69]. However, the prevalence of CKD‐related microvascular calcification is likely to be underestimated in routine brain sections. Other studies using thorough sampling and examination of basal ganglia and hippocampal regions have shown that microvascular calcification is commonly seen in the brains of aged individuals [74] as well as patients with familial CAA [75, 76]; these findings indicate that microvascular calcification is common in MVD and may be clinically significant. One study proposed that adventitial mesenchymal stem cell‐like cells are progenitors of SMC and responsible for driving arterial calcification in CKD [77]. The evidence suggests that calcium and phosphorus metabolism within microvessels plays a key role in vascular abnormalities in CKD‐related MVD, which warrants further investigation in both preclinical and clinical studies.

## CADASIL

CADASIL is an MVD caused by mutations in the *NOTCH3* gene on chromosome 19. CADASIL is the most common genetic cause of stroke. It affects a wide range of age groups, with an early onset in the late 30s [78]. Neuropathological features of CADASIL consist of lacunar infarcts within subcortical white matter, deposition of granular osmiophilic material (GOM) in the media and adventitia of arterioles in both white and grey matter (but especially in the white matter), fibrosis and stenosis of small penetrating arterioles and cerebral microhaemorrhages [79, 80, 81, 82]. GOM can also be found in cutaneous arterioles, a finding which has been used as a diagnostic test for CADASIL; this has largely been superseded by genetic testing [83]. The major components of GOM include the NOTCH3 ectodomain and extracellular matrix proteins. GOM deposition can progress over time, exhibiting alterations in number, size and morphology [84].

**FIGURE 5:**
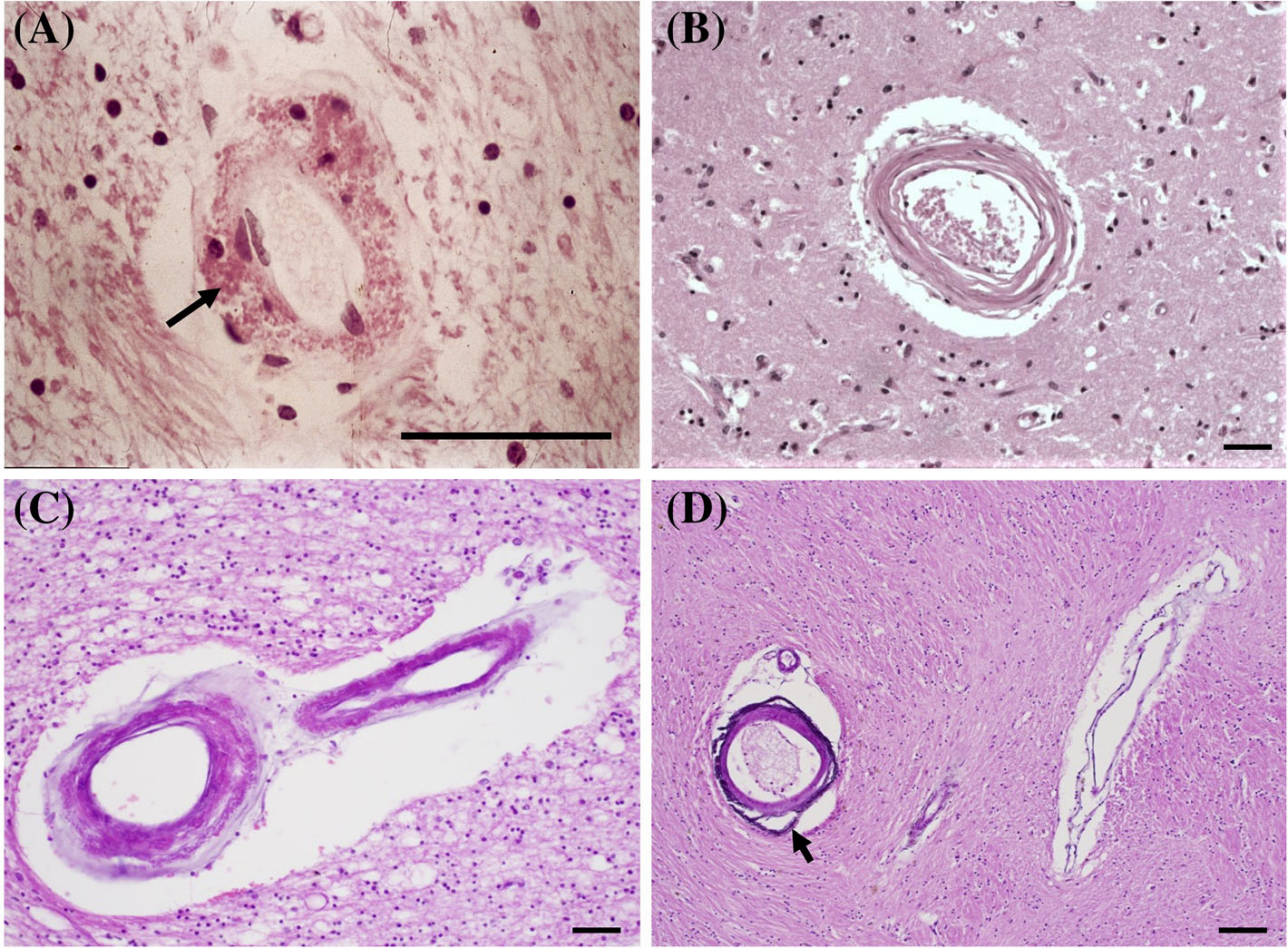
Arteriolar neuropathology in cerebral autosomal dominant arteriopathy with subcortical infarcts and leukoencephalopathy (CADASIL) I. (A) Disruption of proteolipids in arterial vessel walls in CADASIL, with abnormal fat globules (arrow, Oil‐Red‐O). (B) Concentric lamination or onionskin‐type thickening of the arteriolar wall in the basal ganglia of a 30‐year‐old CADASIL subject (hematoxylin & eosin [H&E]). Onionskin‐type thickening of vessel walls is also characteristic of severe hypertensive states often referred to as hyperplastic arteriolosclerosis. (C) Profound vessel wall thickening and glycogen‐cerebroside derangement in CADASIL (H&E). (D) Loss of smooth muscle cells in the media and calcification of arterial walls in the adventitia (arrow) in the basal ganglia in CADASIL (H&E). (A, C) Scale bar 50 μm. (B, D) Scale bar 100 μm

**FIGURE 6:**
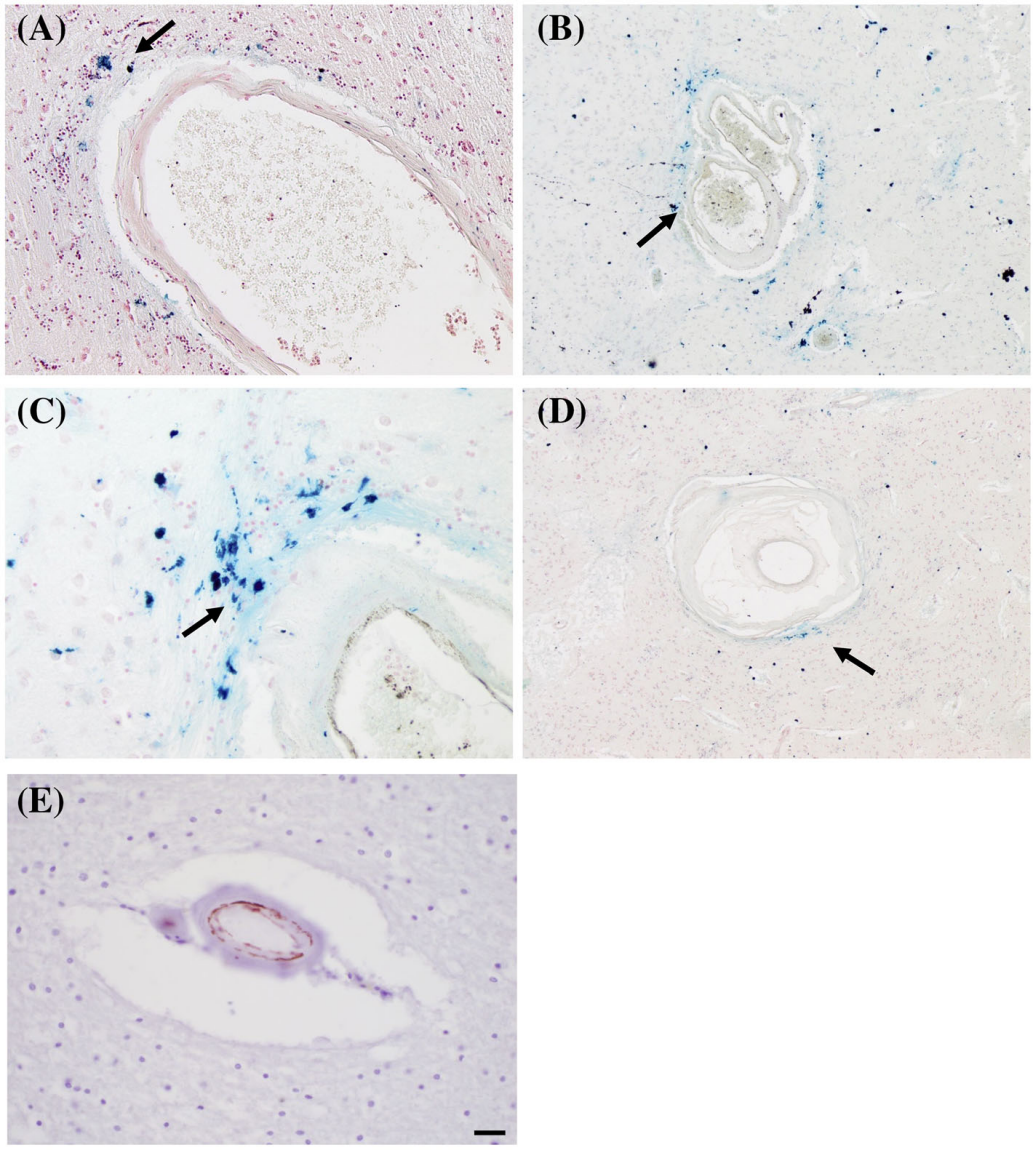
Arteriolar changes in cerebral autosomal dominant arteriopathy with subcortical infarcts and leukoencephalopathy (CADASIL) II. Post‐mortem CADASIL brain shows perivascular haemosiderin‐laden macrophages (arrows), highlighted by Prussian blue staining, adjacent to arterioles in (A) the basal ganglia and (B, C) thalamus. (D) Haemosiderin‐laden macrophages (arrow) adjacent to a basal ganglia vessel with prominent vessel wall thickening. (E) Profound loss of smooth muscle cells in the media, demonstrated by immunohistochemistry for α‐smooth muscle actin, and adventitial thickening in white matter vessels. (A) Magnification ×100. (B) Magnification ×40. (C) Magnification ×200. (D) Magnification ×40. (E) Scale bar 20 μm

*NOTCH3* encodes a transmembrane receptor highly expressed by SMC and pericytes in blood vessels. Over 280 distinct *NOTCH3* mutations cause CADASIL and result in the extracellular domain of *NOTCH3* accumulating in the walls of arterioles, with the diagnostic pathological feature of GOM in blood vessel walls seen on electron microscopy [79, 81]. This leads to degeneration and loss of SMC and fibrosis and stenosis of the small penetrating arterioles [79, 85, 86]. In addition, arteriolar and capillary pericyte degeneration or deficiency appears to contribute to general mural cell loss in the disease [17]. Post‐mortem examination of CADASIL human brain demonstrates disruption of proteolipids in arterial vessel walls (Figure 5A). Other histologic features include vessel wall thickening, concentric lamination or onion‐skinning and hyalinization of vessel walls accompanied by SMC loss and collagen replacement in the media (Figure 5B–D). Perivascular haemosiderin‐laden macrophages are highlighted by Prussian blue staining (Figure 6A–C) adjacent to arterioles, which are characterised by thickened vessel walls and SMC loss in the white matter (Figure 6D–E). Previous studies have demonstrated that SMC from CADASIL patients exhibit reduced proliferation and increased apoptosis compared with those from healthy controls [87, 88]. Mouse models of CADASIL, including *NOTCH3* knock‐out and mutant mice, have been developed to investigate the pathogenic mechanisms underlying CADASIL and test novel therapeutic approaches. These mouse models display various levels of structural abnormalities in cerebral arteries/arterioles and functional impairment in cerebral autoregulation, and may provide essential evidence of the clinical heterogeneity and therapeutic challenges in CADASIL [89].

## CARASIL

CARASIL is a rare, recessively inherited MVD that shares a number of clinical and pathological features with CADASIL, but CARASIL has an earlier onset of cognitive decline with more severe memory impairment. Mutations in high‐temperature requirement A serine peptidase 1 (*HTRA1*) have been linked to the pathogenesis of CARASIL, likely via interfering with TGF‐β signalling [82]. In patients carrying *HTRA1* mutations, abnormal hyperintensities are seen in deep white matter extending from the periventricular to the juxtacortical region with preservation of U‐fibres on brain MRI. Multiple lacunar infarcts primarily detected in the basal ganglia and thalamus are another typical MRI finding. Recent evidence indicates multiple CMB are present in the cortex and white matter of CARASIL patients. The ‘arc sign’ (arc‐shaped hyperintense lesions of the pons and cerebellar peduncles), which may represent pontocerebellar tract involvement, is a characteristic MRI finding in patients with advanced CARASIL [90, 91].

Compared with CADASIL, which is also characterised by affected vessels in a number of organs outside the central nervous system (CNS), vascular pathologic changes in CARASIL are relatively restricted to cerebral arteries and do not show GOM deposition; in addition, non‐vascular extra‐CNS disease may be present [92]. Post‐mortem examination of CARASIL brains revealed arteriolosclerotic changes of small penetrating arteries/arterioles, primarily in the cerebral white matter and basal ganglia, including marked medial and adventitial thinning and degeneration with medial SMC loss. This precedes the development of intimal thickening and fibrosis along with fragmentation of internal elastic lamina (SMC in the thickened intima are also known as ‘myointimal cells’). Due to the loss of SMC and connective tissue in the media (shown by Weigert's elastin and α‐smooth muscle actin immunostaining), the internal elastic lamina seems to be in direct contact with the adventitia and causes a ‘double‐barrelling’ appearance in the vessel wall. As a result, luminal dilatation rather than narrowing is the most frequent finding, especially in the arteries/arterioles located in the leptomeninges and subcortical white matter. In contrast, intimal thickening and narrowing are more frequently seen in the basal ganglia and are associated with stenosis and occlusion [82, 93].

Medial SMC loss is suggested to be the primary mechanism underlying the pathogenesis of CARASIL. First, medial SMC loss is widespread in affected cerebral arteries/arterioles of CARASIL patients, regardless of the presence of sclerotic changes [86, 93]. Second, medial SMC loss may cause myelin damage and ischaemic changes via impaired autoregulation rather than luminal stenosis and adventitial fibrosis [93, 94]. A study combining a mouse model expressing the L364P mutant of the human *HTRA1* gene and primary cell culture suggests that CARASIL induces SMC loss by activating apoptosis signalling [95].

## FABRY DISEASE

**FIGURE 7:**
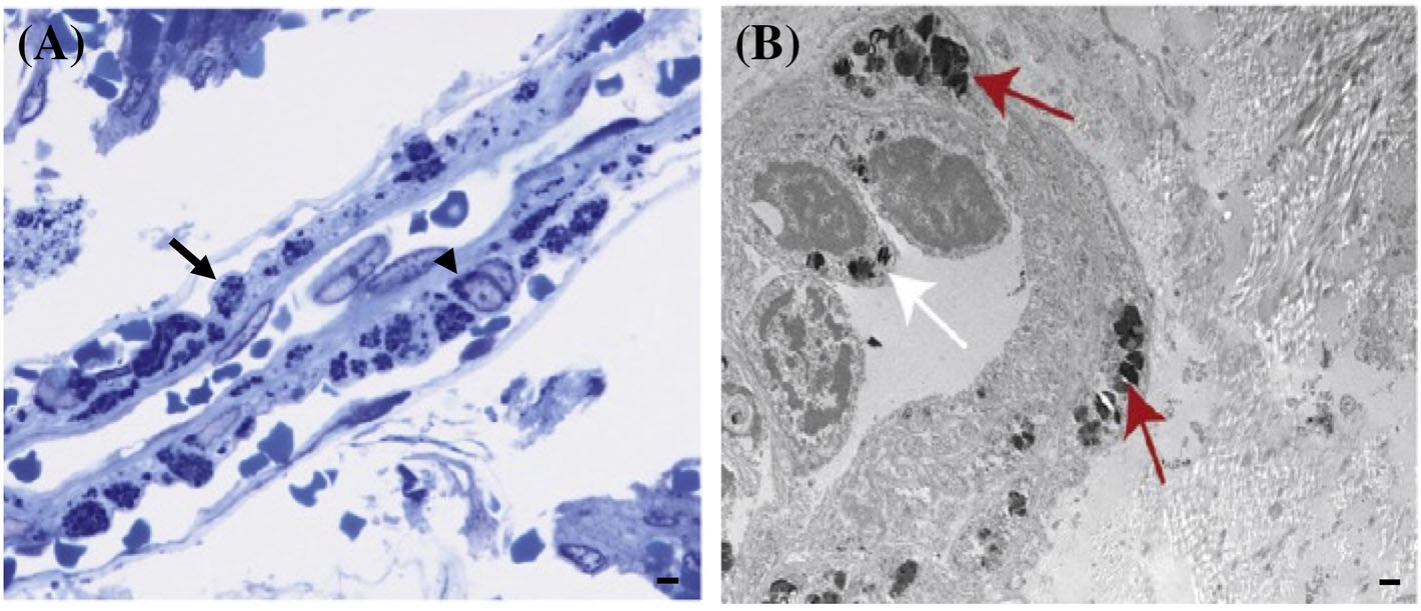
Arteriolar neuropathology in Fabry disease. Lysosomal globotriaosylceramide‐3 (GL‐3) accumulation in vascular cells (arrow: smooth muscle cell [SMC]; arrowhead: endothelial cells) of a 41‐year‐old patient with Fabry disease, with a section taken from a resected meningioma. (A) High‐resolution light microscopy (Richardson's staining). (B) Electron microscopy demonstrates GL‐3 accumulation in SMC (red arrows) and endothelial cells (white arrow). (A, B) Scale bar 1 μm. *Source*: Reprinted from Thurberg et al. Mol Genet Metab Rep 2016;11:75–80, with permission from Elsevier

Fabry disease is an X‐linked hereditary disorder characterised by the accumulation of globotriaosylceramide‐3 (GL‐3) in lysosomes, as a result of a mutation in the *GLA* gene leading to absent or deficient α‐galactosidase A enzyme activity [22]. Ischaemic stroke and transient ischaemic attacks are the most common CNS manifestations in affected patients [96, 97]. Growing evidence suggests that cerebral MVD is the predominant neuropathology in patients with Fabry disease; WMH are the primary feature, followed by CMB and lacunes [98]. Cerebrovascular changes seen in neuroimaging studies include multifocal lesions in the subcortical, deep and/or periventricular white matter and the subcortical, deep grey matter symmetrically in both cerebral hemispheres. Ageing is the primary risk factor that affects the lesion load and pattern of distribution; these lesions may precede the onset of neurological symptoms. A higher white matter lesion load is associated with progression of other cerebrovascular abnormalities. Vascular changes include medial thickening and intimal and adventitial fibrosis [99]. Histological examination reveals extensive lysosomal GL‐3 storage in vascular cells (SMC and endothelial cells) in Fabry patients (Figure 7) [28].

The pathogenic mechanisms of vasculopathy in Fabry disease involve an increase in vessel wall thickness associated with SMC pathology that is distinct from other disease entities. SMC proliferation is considered the major mechanism of increased intima‐media (IM) thickness in Fabry disease and occurs as a result of GL‐3 accumulation in the SMC. Concomitant left ventricular hypertrophy and increased common carotid artery IM thickness in Fabry patients suggest a circulating proliferative factor involved in the vascular pathogenesis. Moreover, SMC proliferation is positively correlated with carotid IM thickness in Fabry patients [100]. In vitro studies show that exposure of SMC to GL‐3 at concentrations observed in the plasma of Fabry patients induce SMC proliferation [101]. A potent proliferative factor sphingosine‐1 phosphate (S1P) in plasma has been identified to be partially responsible for vascular remodelling in this disease. Specifically, higher plasma levels of S1P are seen in Fabry patients compared with healthy controls. Treatment with S1P increase IM thickness in murine aortas and induce SMC proliferation in a dose‐dependent manner [102]. SMC changes in Fabry disease are also associated with increased levels of oxidative stress [103], which may further exacerbate endothelial dysfunction. High circulating levels of MMP (especially MMP‐9) in Fabry disease [104] are linked to deleterious effects on the cerebral arterioles, potentially causing internal elastic lamina degradation and migration of SMC from the media to the intimal layer [105].

## CEREBRORETINAL VASCULOPATHY (CRV) OR RETINAL VASCULOPATHY WITH CEREBRAL LEUKODYSTROPHY (RVCL)

CRV or RVCL is an autosomal‐dominant MVD caused by mutations in the carboxyl‐terminus of three prime exonuclease‐1 (*TREX1*), producing visual impairment and other neurological symptoms. Neuropathologic manifestations include microinfarcts in the white matter and periventricular deep grey nuclei. Granular calcification in the vascular wall is an important characteristic of CRV/RVCL brains with advanced lesions [23, 106, 107].

Ultrastructural examination reveals a thick, multi‐laminated basement membrane within larger vessels, or a single lamina densa within arterioles and capillaries [23]. Other histopathological features include vascular wall thickening and hyalinization, luminal narrowing, adventitial fibrosis and in some cases fibrinoid necrosis [23, 107, 108]. Occasionally, inflammatory cells (CD68^+^ and CD45^+^) can be seen surrounding vessels with intact SMC and endothelium and less fibrosis, whereas fewer SMC and inflammatory cells are observed in the regions bordering ischaemia [23]. A progressive loss of small blood vessels has been seen, but the exact effect of *TREX1* mutation on SMC, and the role of SMC in CRV/RVCL‐related vasculopathy, remains largely unknown. One study identified microRNA (miR)‐103 as a potent regulator of vascular apoptosis, oxidative stress and angiogenesis via targeting *TREX1* in vitro, and its expression was also upregulated in stressed human SMC [109]. This evidence suggests a possible alteration in SMC number in the presence of *TREX1*, which needs to be confirmed in both preclinical and clinical investigations.

## COLLAGEN 4 (COL4) MUTATIONS

Mutations in *COL4*, an essential component of the vascular basement membrane, have been increasingly recognised as risk factors for both sporadic and hereditary forms of MVD. Common variation in the genomic region of *COL4A1/COL4A2*, which encodes the collagen IV chains α1 and α2, is believed to be associated with the sporadic form of deep ICH [110]. Pontine autosomal dominant microangiopathy with leukoencephalopathy (PADMAL) and Swedish multi‐infarct dementia (MID) were previously described as CADASIL‐like disorders but in recent years have been identified to be COL4 disorders [111, 112, 113, 114]. PADMAL can be caused by two types of *COL4A1* mutation leading to different neurological events. Glycine missense mutations in *COL4A1* may predispose to cerebral haemorrhage, whereas mutations upregulating *COL4A1* gene expression are associated with ischaemic strokes [114]. Patients exhibit recurrent ischaemic and haemorrhagic strokes between age 35 and 45 years that are associated with progressive imbalance and cognitive impairment but generally no additional neurological impairment. Dominant mutations in *COL4A1/COL4A2* occur within the conserved glycine‐X‐Y motifs in the triple‐helical collagenous domain involving glycine substitutions, whereas those in PADMAL and MID disorders occur in the 3′ untranslated region of *COL4A1*. Dominant mutations in *COL4A1/COL4A2* can cause a monogenic form of MVD, manifesting as lacunar infarction, WMH and haemorrhagic lesions ranging from asymptomatic CMB to life‐threatening large deep haemorrhages [115, 116]. In COL4‐related MVD, cerebral microhaemorrhages and macrohaemorrhages are age‐related and believed to originate from different vascular sources that reflect distinct stages of the vascular disease; this may affect subsequent therapeutic interventions. In a mouse model expressing the *COL4A1* Gly498Val mutation, numerous microhaemorrhages were detected early in life and were associated with BBB disruption. Changes in the vessel wall surrounding macrohaemorrhages included focal loss and fragmentation of SMC and elastin in the medial layer, leaving endothelial cells in direct contact with the parenchyma; the remaining SMC exhibited an abnormally thin, discontinuous morphology without dense plaques [117].

**FIGURE 8:**
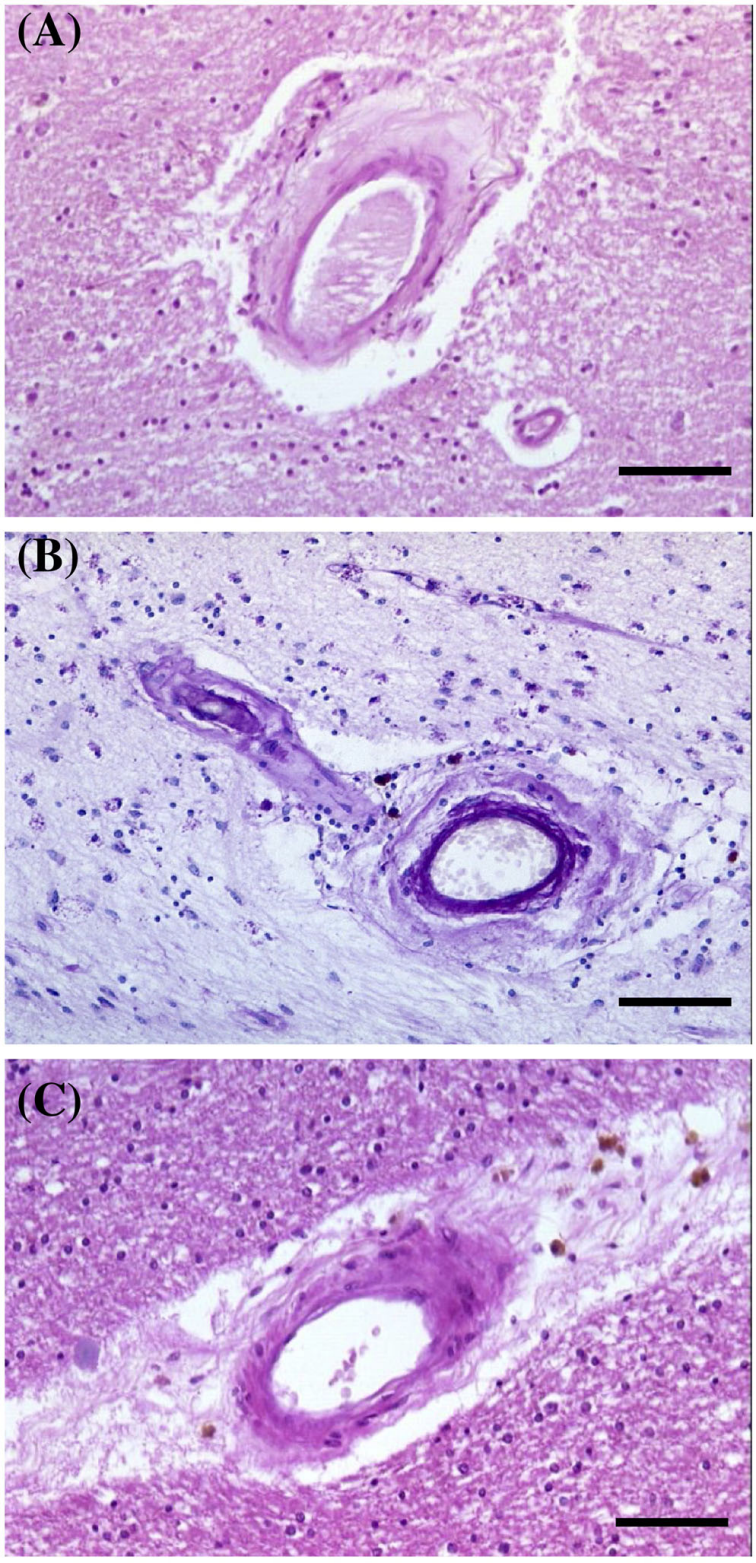
Arteriolar neuropathology in collagen IV (COL4) mutations. (A) Differential changes in arteriolar walls in COL4 arteriopathy with COL4A1 c.*32G>A mutation in the 3′ untranslated region. (B) Cortical vessel with severe smooth muscle cell (SMC) loss and fibrosis in the adventitia in a 60‐year‐old patient. Periodic acid‐Schiff (PAS)‐positive arteriolar walls also found in COL4A1 arteriopathy in the cortex. (C) White matter vessel with mild–moderate loss of SMC in a 30‐year‐old patient with intracerebral haemorrhage. (A–C) Scale bar 100 μm

Accumulating evidence suggests the central role of SMC in the pathogenic mechanisms underlying COL4‐related MVD [117, 118, 119, 120]. Our histological examinations of COL4 arteriopathy in patients with *COL4A1* mutation (c.*32G>A) reveal differential changes in arteriolar walls with prominent SMC loss (Figure 8). Vascular basement membrane composed of collagen IV, laminin and heparan sulphate proteoglycans forms a three‐dimensional protein network to support interactions between SMC with other cellular components [121]. *COL4A1/COL4A2* mutations exert effects on SMC behaviour via multiple mechanisms, including inducing SMC apoptosis [117, 119], impairing SMC differentiation and maintenance via disruption of vascular basement membrane [118] and promoting the production of a pathogenic, synthetic phenotype of SMC via binding to cellular receptors [120]; these can all cause detrimental effects on vessel wall integrity and function.

## CARASAL

In addition to the classic genetic factors (i.e., *NOTCH 3*, *HTRA1*, *GLA*, *TREX1* and *COLA1/A2*), it has been shown that mutations in the *CTSA* gene encoding cathepsin A can cause a novel MVD, namely CARASAL. This can present clinically with ischaemic stroke, cognitive impairment and therapy‐resistant hypertension [122, 123]. Neuropathological examination shows mild white matter atrophy and small infarcts dispersed widely in the brain, including white matter, deep grey matter, brainstem and cerebellum. This is accompanied by changes in cerebral arterioles including asymmetric fibrous thickening, loss and degeneration of medial SMC, varying degrees of luminal narrowing from stenosis in small arterioles to total occlusion in large arterioles and enlarged adventitia with deposition of collagen fibrils [124]. Homozygous *CTSA* mutation is thought to cause CARASAL by interfering with the function of cathepsin A, which protects against a systemic lysosomal storage disorder by stabilising the lysosomal enzymes β‐galactosidase and neuraminidase [124]. Heterozygous *CTSA* mutation might alter cathepsin A activity in inactivating endothelin‐1, therefore impairing blood pressure regulation [124].

## CONCLUSIONS

There are many cerebral MVD entities but only a limited range of parenchymal consequences of these disorders. Cerebral MVD has both ischaemic and haemorrhagic components with neuropathology that ranges from lacunar infarcts, microinfarcts and white matter disease to microhaemorrhages. MVD is consistently characterised by substantial arteriolar remodelling (cerebral angiomyopathy) involving alterations in SMC via proliferation, apoptosis, phenotypic switch and/or migration with resultant changes in vessel wall components, diameter and thickness (Table 1). Taken together, these elements strongly support the arteriole acting as both source and mediator of parenchymal injury. Thus, it is the arteriole that is the critical component and final common pathway of cerebral MVD.

**TABLE 1:** Summary of arteriolar neuropathology associated with multiple risk factors and disease entities

Risk factors and disease entities	Prevalence	Intima	Media	Adventitia	References
Ageing	Universal	Intimal thickening evident by a decrease in lumen diameter and an increase in intima‐to‐media thickness Multilayered intima due to smooth muscle cell (SMC) migration from the media to intima and infiltration of inflammatory cells Endothelial dysfunction	Medial elastin loss with replacement by collagen Inconsistent findings on SMC proliferation: ○ Increased SMC proliferation (increased expression of stem cell and cell cycle activation markers) ○ Loss of proliferation and increased apoptosis with advanced age SMC phenotypic switch and migration from the media to the intima: ○ Accelerated cell cycle ○ Increased reactive oxygen species (ROS) and matrix metalloproteinase (MMP) production	Adventitial fibrosis	[7, 24, 30, 31, 32, 33, 34, 35, 36, 37, 38, 39, 40, 41, 42, 43, 44]
Hypertension	Common	Intimal thickening Endothelial dysfunction	Arteriolosclerosis: arteriolar wall thickening with SMC degeneration and loss Hyalinosis: the affected vessel wall (typically less than 150 μm in diameter) can develop a ‘glassy’ hyalinized and/or an ‘onion‐skinning’ hyperplastic appearance, from degenerated SMC and elastin in the media and proliferated fibroblasts in the adventitia. Fibrinoid necrosis: infiltration of fibrin and fibrin degradation products in the vessel wall Microatheroma: distal manifestations of atherosclerosis involving larger arterioles Microaneurysms: segmental dilatation of vessels Proliferation of mural cells may exert secondary effects that promote SMC degeneration in the upstream feeding arterioles.	Adventitial fibrosis and extracellular matrix (ECM) remodelling	[2, 9, 11, 46, 47, 48, 49, 50]
Cerebral amyloid angiopathy (CAA)	Sporadic CAA: common Hereditary CAA: rare	Endothelium is well‐preserved regardless of CAA progression and severity. The internal basal lamina appears thinner and irregular as CAA progresses with advanced amyloid‐β (Aβ) deposition.	Early stage of CAA: Aβ deposition in the external basal lamina in close proximity to the SMC layer; no SMC replacement or disruption Advanced stage of CAA: Aβ deposition extends to the SMC layer, followed by SMC loss and complete replacement by Aβ. This stage is also associated with microaneurysms and fibrinoid necrosis.	Accumulation of Aβ in the adventitial layer in the early stage of CAA	[58, 59, 60, 61, 62]
Chronic kidney disease (CKD)	Common	Intimal thickening Endothelial dysfunction	Hyalinization and arterial/arteriolar wall thickening: deposition of collagen and connective tissues Early stage of CKD: hyperphosphatemia induces apoptosis of SMC. Late stage of CKD: hyperphosphatemia causes further weakening of the vessel wall and arterial calcification by inducing an osteogenic phenotype shift of SMC and elastin degradation.	Adventitial fibrosis; adventitial mesenchymal stem cell‐like cells are progenitors of SMC and responsible for driving arterial calcification in CKD.	[67, 68, 69, 70, 71, 72, 73, 74, 75, 76, 77]
Cerebral autosomal dominant arteriopathy with subcortical infarcts and leukoencephalopathy (CADASIL)	Rare	Intimal thickening Endothelial dysfunction	Medial thickening with accumulation of debris from SMC degeneration or loss, as well as collagen and granular osmiophilic material (GOM) deposition in small penetrating arterioles Reduced proliferation and increased apoptosis of SMC	Adventitial fibrosis	[17, 79, 80, 81, 82, 83, 84, 85, 86, 87, 88, 89]
Cerebral autosomal recessive arteriopathy with subcortical infarcts and leukoencephalopathy (CARASIL)	Very rare	Intimal thickening and fibrosis along with fragmentation of internal elastic lamina SMC with scattered distribution in the thickened intima are known as ‘myointimal cells’. Luminal dilatation in the arteries/arterioles in the leptomeninges and subcortical white matter Intimal thickening and narrowing in the arteries/arterioles in the basal ganglia	Medial thinning and degeneration with SMC loss are primary findings and precede intimal changes. Due to the loss of SMC and connective tissues in the media, the internal elastic lamina seems to be in direct contact with the adventitia and causes a ‘double‐barreling’ appearance in the vessel wall.	Adventitial thinning and degeneration	[82, 86, 92, 93, 94, 95]
Fabry disease	Rare	Intimal thickening Endothelial dysfunction due to globotriaosylceramide‐3 (GL‐3) accumulation in the vascular endothelial cells	Medial thickening with increased SMC proliferation as a result of GL‐3 accumulation in the SMC A proliferative factor sphingosine‐1 phosphate (S1P) in plasma may be responsible for increased SMC proliferation. High level of MMP‐9 leads to migration of SMC from the media to the intima.	Adventitial fibrosis	[28, 99, 100, 101, 102, 103, 104, 105]
Cerebroretinal vasculopathy (CRV) or retinal vasculopathy with cerebral leukodystrophy (RVCL)	Very rare	A thick, multi‐laminated basement membrane within larger vessels, or a single lamina densa within arterioles and capillaries Endothelial changes: ○ Intact endothelial layer in vessels with less fibrosis ○ Endothelial dysfunction within regions of ischemia	Vascular wall thickening and hyalinization Fibrinoid necrosis SMC changes: ○ Intact smooth muscle layer in vessels with less fibrosis ○ Fewer SMC in the regions bordering ischemia ○ No SMC within regions of ischemia	Adventitial fibrosis	[23, 106, 107, 108, 109]
Collagen IV (COL4) mutations	Rare	Endothelial cells seem to be in direct contact with the parenchyma due to medial SMC loss and fragmentation	Focal loss and fragmentation of SMC and elastin in arteries/arterioles producing macrohaemorrhages Remaining SMC exhibit an abnormally thin, discontinuous morphology without dense plaques.	Variable adventitial fibrosis	[117, 118, 119, 120, 121]
Cathepsin A‐related arteriopathy with stroke and leukoencephalopathy (CARASAL)	Extremely rare	Asymmetric fibrous thickening Varying degrees of luminal narrowing from stenosis in small arterioles to total occlusion in large arterioles	Loss and degeneration of medial SMC in arteries/arterioles	Enlarged adventitia with deposition of collagen fibrils	[122, 123, 124]

## CONFLICT OF INTEREST

The author(s) declared no potential conflicts of interest with respect to the research, authorship and/or publication of this article.

## AUTHOR CONTRIBUTIONS

*Manuscript design, literature search and manuscript drafting and revision*: Chuo Fang. *Manuscript drafting and revision*: Shino D. Magaki. *Manuscript drafting and revision*: Ronald C. Kim. *Manuscript drafting and revision*; Raj N. Kalaria. *Manuscript drafting and revision*: Harry V. Vinters. *Manuscript design, literature search and manuscript drafting and revision*: Mark Fisher.

## Data Availability

Not applicable.
